# The Impact of Uterus Didelphys on Fertility and Pregnancy

**DOI:** 10.3390/ijerph191710571

**Published:** 2022-08-25

**Authors:** Adrianna Ćwiertnia, Dominika Borzyszkowska, Anna Golara, Natalia Tuczyńska, Mateusz Kozłowski, Sebastian Kwiatkowski, Aneta Cymbaluk-Płoska

**Affiliations:** 1 Department of Gynecological Surgery and Gynecological Oncology of Adults and Adolescents, Pomeranian Medical University, 70-111 Szczecin, Poland; 2 Department of Obstetrics and Gynecology, Pomeranian Medical University, 70-111 Szczecin, Poland

**Keywords:** uterus didelphys, fertility, pregnancy, uterine malformation, Müller’s duct anomaly

## Abstract

Uterus didelphys occurs as a result of abnormal fusion of the paramesonephric ducts and is characterized by complete duplication of uterine horns, cervix, and very often also the vagina or presence of longitudinal vaginal septum. Most women with a uterus didelphys are asymptomatic; some cases may coincide with dyspareunia or dysmenorrhea. The anomaly is associated with a higher risk of miscarriage, preterm labor, breech delivery, and decreased live births. We present the case of a 26-year-old woman (primigravida) who was known to have uterus didelphys. The diagnosis was made when the patient was 23 years old using ultrasound and hysteroscopy. The patient became pregnant after 18 months of efforts. The patient was referred to prenatal care in the 13th week of pregnancy with vaginal bleeding. In the 23rd week of pregnancy, gestation cholelithiasis was diagnosed. The pregnancy progressed without obstetric complications and the fetus developed normally. Due to the vaginal septum and fact that the patient felt stressed, the pregnancy was terminated at term by cesarean section. We concluded that uterus didelphys can be asymptomatic making an early diagnosis difficult. A pregnancy belongs to a high-risk group and more attention should be paid to this case. Cesarean section should be considered, especially in case of the presence of vaginal septum.

## 1. Introduction

Uterine malformations arise as a result of abnormal formation, fusion, or resorption of the Müllerian ducts during fetal life. Reproductive organ malformations occur in approximately 4.3% of fertile women and approximately 3.5% in infertile women, and the uterine defect that contributes most to infertility is the unicornuate uterus. The most common defects of the reproductive organ are septate uterus (approximately 35%) and bicornuate uterus (approximately 25%) [1]. In contrast, uterus didelphys is one of the rarest, accounting for 10% of all Müller’s duct anomalies [2]. Uterus didelphys arises from incomplete fusion of Müller’s ducts between 12 and 16 weeks of fetal life, followed by a dilation of the uterine horns, cervix and, very often, the vagina [3]. The clinical course of the defect is asymptomatic in the majority of patients, which contributes to the diagnosis being made only at reproductive age. However, it is sometimes manifested by dyspareunia and/or painful menstruation [4]. The presence of a uterine defect increases the risk of obstetric complications, indicating the need for frequent checks during pregnancy. Above all, there is an increased risk of spontaneous miscarriage, preterm births, births in the breech position, and a reduced number of live births compared to a normal uterus [5]. Preterm births occur in approximately 17.44% to 33.3% of women with uterus didelphys [6]. The diagnosis is made on the basis of imaging studies—ultrasound, HSG, and MRI. Here, we present a clinical case of the patient with uterus didelphys who successfully conceived, carried her pregnancy, and gave birth to a healthy infant by cesarean section.

## 2. Case Report

### 2.1. Gynecological History of the Patient

**Figure 1:**
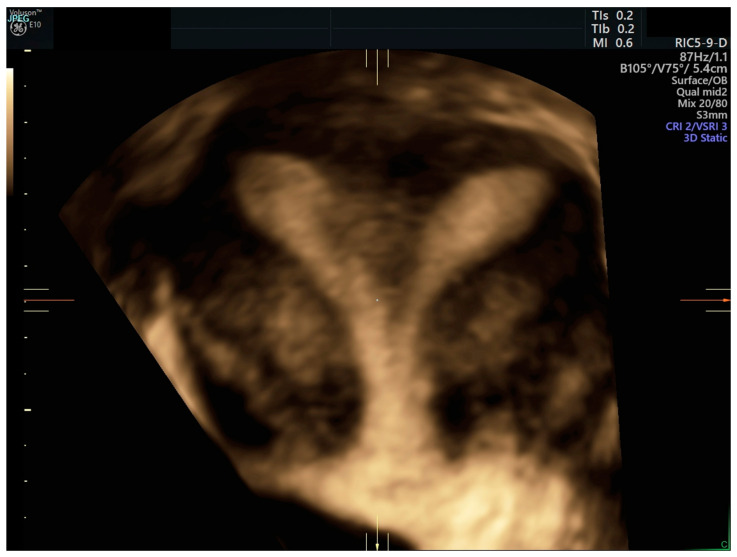
Uterus didelphys showing in 3D ultrasound mode.

A 23-year-old female patient admitted herself to the Department of Gynecological Surgery and Gynecological Oncology of Adults and Adolescents, Pomeranian Medical University in Szczecin for a palpable vaginal membrane. The patient denied the presence of dyspareunia or dysmenorrhea. A transvaginal ultrasound was performed, which allowed a preliminary diagnosis to be made—uterus didelphys (Figure 1). 

Confirmation of the diagnosis was made possible by hysteroscopy, during which a double uterus, two cervixes, and an elongated septum running through half of the vagina were visualized. 

The patient reached menarche at the age of 13 years. In the patient’s teenage years hypomenorrhea occurred which resolved spontaneously. The patient had been using combined oral contraceptives (ethinylestradiol + norgestimate) for 6 years. She discontinued the pills in order to get pregnant which took approximately 18 months.

### 2.2. Infertility and Partner

The patient and her partner had been trying to have a baby for 18 months. Regular sexual intercourse was accompanied by psychological discomfort. The patient was concerned about the defect, keeping in mind the double uterus, worrying about the future and chances to become pregnant. In the patient’s hormonal tests, no irregularities were found apart from prolactin that was slightly elevated (23.70 ng/mL; ULN: 23.30 ng/mL), but no clinical symptoms occurred. According to the recommendations of PSRME (Polish Society of Reproductive Medicine and Embryology) and PSGO (Polish Society of Gynecologists and Obstetricians), diagnosis of the cause of infertility should be performed in both partners. For this reason, the patient’s partner underwent diagnostics. Laboratory tests showed increased prolactin levels (48.70 ng/mL; ULN: 15.20 ng/mL), the rest of the hormonal tests (testosterone, thyroid-stimulating hormone, follicle-stimulating hormone, luteinizing hormone, estradiol, progesterone, cortisol, and β-subunit of human chorionic gonadotropin) were at normal range. Semen analysis showed oligoasthenoteratozoospermia (irregular sperm count, mobility, and morphology with reduced vitality). About 8 months after hormonal diagnostics, he underwent laparoscopic embolization of varicocele with indication: an incorrect result of the semogram.

### 2.3. Pregnancy

Transabdominal ultrasound examination of the first trimester visualized a viable fetus in the left uterus. 

In the general evaluation, cardiac function, triovascular structure of the umbilical cord, and a normal amount of amniotic fluid were visualized in this examination the fetal anatomy was visualized without abnormalities. Doppler examination showed the following pulsation indices: in the left uterine artery, 1.73 and in the right uterine artery, 1.22. Tricuspid flow was proper, and no other markers of chromosomal aberrations were found. Measurements of free β-hCG (β-subunit of human chorionic gonadotropin) and PAPP-A (Pregnancy-associated plasma protein A) showed a low risk of Down, Edwards, and Patau syndromes. An ultrasound performed in the second trimester showed normal fetal development and anatomy. A compatible gestational age and normal growth of biometric parameters were found. Fetus in cephalic presentation, movements present, heart rate (FHR) 150 bpm. Weight was estimated at 347 g. Anterior placenta, amniotic fluid volume normal, and normal three-vessel umbilical cord. Blood flows in the ductus venosus, uterine, and umbilical artery were normal. 

At 13 weeks of pregnancy, the patient presented with sudden gynecological hemorrhages that resolved after one month treating with dydrogesterone and ethamsylate. In the 23rd week of pregnancy the patient experienced a severe pain in the upper abdomen. Laboratory results showed increased serum amylase activity (169 U/L). During transabdominal ultrasound sonography, the patient was diagnosed with cholelithiasis. Laparoscopic cholecystectomy is one of the possible ways of treatment for cholelithiasis and was considered in this case. However, after fluid infusion, paracetamol i.v (intravenous), drotaverine i.v., lignocaine i.v., and progesterone per vaginam symptoms subsided. Afterwards, laboratory results showed decreased serum amylase activity. The cervix was not changed. USG showed fetal eutrophy and well-being. 

### 2.4. Labor

The patient was admitted to the Department of Obstetrics and Gynecology, Pomeranian Medical University in Szczecin, with a term pregnancy (40 + 1 Hbd). On admission, the general condition was good, and blood pressure, temperature, and pulse were normal. She did not report any additional complaints. Laboratory tests for infections: GBS, HBV, Treponema, and HIV were negative. On gynecological examination, discharge was normal, and vaginal part of the left cervix 0.5/2/2. Fetal position was longitudinal cephalic. Due to the double uterus with vaginal septum and concerns from the patient, the surgical team decided to terminate the pregnancy by cesarean section, to which the pregnant woman consented. A newborn baby was extracted behind the head from clear amniotic waters. The afterbirth was removed by pulling on the umbilical cord. The uterine cavity was curetted on both sides of the septum. On postoperative day 2, the obstetrician’s general condition was good, respiratory rate, heart rate, and temperature were normal, abdomen soft non-painful, peristalsis preserved, and diuresis was normal. The operative wound was dry. The uterus was contracted, uterine cavity discharge mediocre, serous-bloody, typical, not stinking. The patient did not report any complaints.

The male neonate was in good general condition, Apg 10/10, umbilical cord blood pH = 7.37, with a body weight of 3550 g. After birth, there were apical systolic murmur and limb tremors, which resolved on the next day of life. The period of adaptation in the ward proceeded with physiological neonatal jaundice, breastfed on demand. He was discharged together with the patient on day 3 of life in good general condition. Both mother and baby were healthy at discharge.

## 3. Discussion

Diagnosis of uterine malformations is usually problematic and lengthy, due to the asymptomatic course of uterine anomalies. For diagnosis, the following are used: ultrasound, HSG, and MRI. However, the detection of uterine malformations has increased in the last decade thanks to better diagnostic methods. If there are no coexisting symptoms, treatment is waived. On the other hand, in case of obstetric failure, surgical treatment is possible, involving the fusion of two duplicated uteruses. The most widely used classification of uterine malformations is the American Society for Reproductive Medicine (ASRM) classification (updated and expanded American Fertility Society (AFS) classification), which outlines nine main types of uterine anomalies [7]. According to AFS classification, uterus didelphys belongs to class III. ASRM, in their classification, divided the group of uterus didelphys into three: 1. Uterus didelphys and longitudinal septum, 2. uterus didelphys and +/− longitudinal vaginal septum of variable length, and 3. uterus didelphys and obstructed R/L hemivagina [8]. In 2013, European Society of Human Reproduction (ESHRE) and Embryology-European Society for Gynaecological Endoscopy (ESGE) published a new classification [9]. Uterine anomalies are divided into six main groups and subgroups, which also include cervical and vaginal anomalies. ESHRE classifies uterus didelphys as U3—bicorporeal uterus C2—double normal cervix V1—longitudinal non-obstructing vaginal septum [9].

Uterine malformations are most often detected in patients of reproductive age due to fertility problems (previously no symptoms), as many as 2–8% of infertile women have a uterine malformation and 5–30% have a history of miscarriage. Bicornuate, unicornuate, and didelphic uterus are generally not a direct cause of infertility. However, it might be associated with aberrant outcomes throughout the course of pregnancy [10]. Chan et al. suggested that women with canalization defects, such as septate and subseptate uteri, appear to have the poorest reproductive performance, they are at increased risk of first-trimester miscarriage, preterm birth, and fetal malpresentation at delivery [10]. Many uterine defects remain undiagnosed for many years, so Mohamed et al. suggested that there is a significant opportunity to diagnose congenital uterine anomalies during cesarean section within seconds, without increasing operative time and risk to patients [11]. The presence of a uterine defect may be a stress factor for the patients. The detection of this defect at reproductive age can further exacerbate it, due to the awareness of the complications that pregnancy may entail. This may lead to an increased level of stress experienced by patients during pregnancy, particularly just before and during labor. Stress is cited in the literature as one of the risk factors for preterm birth [12]. Natural childbirth accompanied by increased patient stress is fraught with complications. The above case report presents a patient with uterus didelphys who became pregnant after 18 months of trying (what the World Health Organization defines as having sex 2–3 times a week). The complexity of the infertility problem takes into account both the presence of a patient’s uterine defect as well as disturbances in hormonal tests and oligoasthenoteratozoospermia in the patient’s partner. The pregnancy progressed without obstetric complications which allowed the pregnancy to be carried to the scheduled labor date, but after a vaginal bleeding episode in the 13th week of pregnancy and an episode of cholelithiasis in the 23rd week, which further increased anxiety and stress, the patient began to suffer from insomnia. She felt stressed trying to become pregnant and was concerned about the course of the delivery. A condition such as cholelithiasis should be differentiated from uterine rupture, which is extremely rare, but leads to life-threatening complications for both the fetus and the mother. Damiani et al. presented an unexpected UR (uterine rupture) that occurred at 23rd week of pregnancy [13]. The first symptom was abdominal pain of sudden onset. Due to cramping pain in the upper or lower abdomen, UR can be misdiagnosed as gastrointestinal problems. Shi et al. presented the case of a patient who was misdiagnosed with acute appendicitis. [14]. Li et al. described the case of a patient at 16 weeks gestation who was first admitted to the gastroenterology department because of recurrent intermittent lower abdominal pain and vomiting. After that, this patient was admitted to the ward two more times: at 24 and 28 weeks. At 29 weeks and 6 days of gestation, she complained of an acute abdominal pain accompanied by diarrhea and vomiting. In this patient, even though she was nulliparous, the risk of UR was higher due to the presence of adenomyosis at the site of uterine rupture and diamniotic dichorionic twin pregnancy after in vitro fertilization and embryo transfer [15]. The most common risk factor of UR is history of uterine surgery, for example cesarean section and myomectomy. Although uterine rupture is not a condition characteristic of nulliparous woman and in unscarred uterus, patients with uterine malformations should be monitored very thoroughly [13]. The literature shows that any non-specific abdominal pain even in the second trimester of pregnancy should be alarming, and the diagnosis should be made carefully, as delaying it leads to dangerous consequences.

The proper function of the placenta is essential for the adequate progression of the pregnancy until its termination and also translates into perinatal outcome. In the described case of a 26-year-old female patient, the placenta was located on the anterior wall, without features of separation. The pregnant woman’s history does not include diseases affecting the vasculature and thus the uteroplacental circulation. No exponents suggestive of placental insufficiency were observed during pregnancy. The neonate was born in a good general condition and adaptation was normal, without complications. A prospective study by Loverro et al. involving 350 pregnant women and 380 newborns presents a correlation between placental changes and the main neonatal pathologies [16]. Obstetric histories and histological examination of the placenta were compiled. Placental lesions according to the Amsterdam criteria are divided into vascular and inflammatory-immunological [17]. This study shows a significant association between respiratory disorders (61 cases) and generic placental anomalies (*p* = 0.006). The number of cases of neonatal sepsis (15 cases) correlates with placentitis (*p* = 0.035) and villitis of unknown origin. The largest number of cases were preterm births. A possible influence of premature malperfusion on the increased risk of preterm births was noted (*p* = 0.0011). A thorough evaluation of the placenta can provide useful information about the prenatal prognosis [16]. If a uterine defect such as uterus didelphys is present, placental function should also be monitored with great care, as uterine and placental abnormalities can increase the risk of preterm births.

Pregnancy with uterus didelphys has an increased risk of preterm birth, so more attention should be paid to these cases. This risk varies between different uterine defects. Khander et al. included 283 women and presented results comparing the risk of preterm birth in women with uterine malformations in unborn women and those already in labor. The risk of preterm birth in unborn women with a major uterine defect (uterus didelphys, unicornuate, bicornuate) is 19.6%, in women with a history of obstetrics 15.9%, and in women who have had a preterm birth 57.1% [18]. Another cohort study shows the risk of preterm birth in individual uterine malformations [6]. Women with a bicornuate uterus have the highest risk of preterm birth with 59.3%, with a septate uterus and a double uterus with 17.44%. For a unicornuate uterus, the risk of preterm birth is 5.81% [6]. In this case, the pregnancy of a 26-year-old primigravida with a double uterus was terminated at the expected date of 40 + 1 Hbd.

Uterus didelphys as an isolated anomaly is not an indication for either surgical treatment or termination of pregnancy by cesarean section. The patient’s reproductive organ defect consists of a double uterus, but also two cervixes and one vagina, however partitioned by an elongated membrane, which was of particular interest for natural childbirth. The restriction of the size of the vagina and its division into two parts may have represented a risk of complications for the newborn and a prolongation of the second phase of labor. Slavchev et al. also described a septum in vagina and suggest that CS is a safer method for this defect of Müller’s ducts and that more in-depth analysis of the data is needed to clearly assess the safety of natural childbirth in uterus didelphys [2]. In contrast, Rezai et al. suggested that didelphys uterus is not an indication for cesarean delivery unless the vaginal septum is thick and inelastic resulting in an increased risk for vaginal dystocia, and our patient had a thin, membranous septum up to half the vaginal length [4]. Table 1 presents data collected from the literature on pregnancies in patients with uterus didelphys in the last 10 years.

A valid aspect is also the fact that the patient experienced fear for the welfare of her child, because she was aware that the uterine defect may affect the child’s development. She was trying to get pregnant for 18 months; at 13 weeks gestation with the onset of sudden vaginal bleeding and with a ductal stone at 26, she was repeatedly exposed to stress, which was increased by the presence of a uterine defect. The patient was even more stressed during the week of delivery, which she related to her concerns about the course of natural childbirth. Recommendations of the Polish Society of Gynecologists and Obstetricians regarding cesarean section (2018) indicate that the group of indications concerns the presence of strong anxiety before childbirth (tocophobia), anxiety reflecting the presence of other types of anxiety disorders, or anxiety occurring in the course of depressive disorders [38]. Goulios et al. highlighted that without strong evidence for either vaginal or cesarean delivery in cephalic presentations, factors that should be considered for pregnancy include the clinical recommendations by the medical team and the mother’s preferences [23]. Clinical complications of uterus didelphys possibly include, but are not limited to breech presentation. This malposition is the main reason for the necessity to terminate the pregnancy by cesarean section [23].

**Figure 2:**
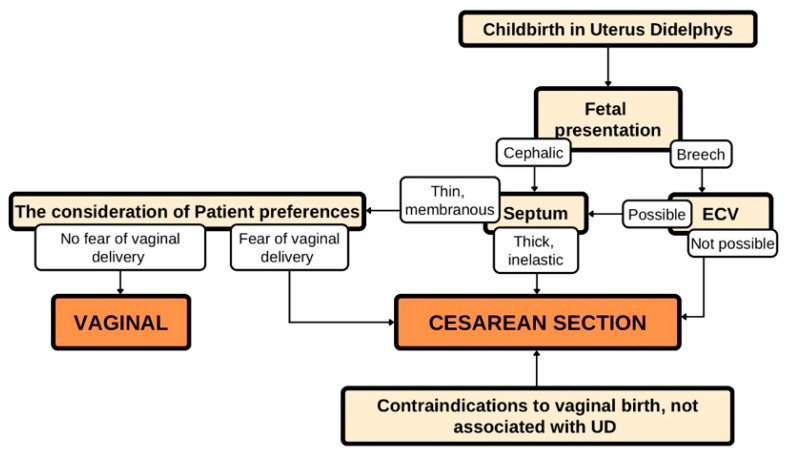
Termination of pregnancy in uterus didelphys depending on various factors.

Frequency of breech presentation in term births was 3–4% [39]. In patients with uterus didelphys, breech position referred to 43% and CS was more common than vaginal delivery [40]. Due to this management, risk of perinatal mortality is lower [41]. Not only CS but also vaginal delivery can be successful when this malposition occurs. Such a unique case was described by Mirzai et al. where vaginal delivery in the patient with uterus didelphys went well after the previous ECV (external cephalic version) [42]. Slavchev et al. described three individual cases of pregnancy in uterus didelphys [2]. The deliveries were accompanied by the cephalic presentation in all three cases. Even though the presentation of fetuses was physiological, two of them were delivered by cesarean section. One, which ended in a vaginal delivery, went without complications; the septum was moved through the descendent part of the fetus [2]. King et al. described a case of a twin pregnancy in which both fetuses were in the cephalic presentation. The first fetus was delivered vaginally, while the second was delivered by cesarean section [21]. In the case of our patient, the fetus also had a cephalic position. At first, termination of pregnancy was qualified for vaginal delivery, as there were no indications for cesarean section. However, due to the strong fear of the patient for the baby and presence of septum in the vagina, the medical team together with the patient decided to perform a cesarean section. The decision to perform a cesarean section in this case requires taking into account many factors, which are presented in the chart below (Figure 2).

## 4. Conclusions

The absence or scant clinical signs of uterine didelphys make early diagnosis difficult. Infertility is not caused by uterus didelphys directly, therefore diagnostics should not be limited to visualizing the defect. It is worth looking at the couple in a holistic way, so the diagnostics of both the woman and the man should be expanded. Focusing only on the uterine defect as a possible cause of infertility may obscure a proper genesis. If uterus didelphys coexists with other medical factors, such as the presence of a septum in the vagina or the patient’s concerns, a cesarean section should be considered. Decision-making emphasizes the priority of proper communication between the medical team and the patient, especially when making key decisions regarding the course of pregnancy and childbirth.

## Figures and Tables

**Table 1:** Cases of pregnant women with uterus didelphys defect from the literature in last 10 years. N/A—not applicable; ND—no data.

Age of Dia-Gnosis	Age of Pre-Gna-Ncy	Presence of a Vaginal Septum	Fetus in the Right/Left Uterus	Position of the Fetus	Natural Childbirth/ Cesarean Section	Indications for Cesarean Section	References
23	26	present	left	longitudinal cephalic	cesarean section	the strong fear of the patient for the baby and presence of septum in the vagina	the case described in this article
35	35	present	right	breech presentation	cesarean section	breech presentation	[19]
28	29	present	left	anterior presentation	natural childbirth	N/A	[4]
20	21	present	left	ND	cesarean section	ND	[2]
25	25	absent	left	frontal occipitoparietal position	cesarean section	no progress in labor	[2]
27	27	present	right	frontal occipitoparietal position	natural childbirth	N/A	[2]
27	35	absent	right	ND	cesarean section	ND	[20]
27	27	absent (removed in the past)	twin pregnancy	ND	1—natural childbirth (right uterus); 2—cesarean section (left uterus)	chorio- amnionitis	[21]
ND	35	ND	twin pregnancy	ND	cesarean section	it was uncertain whether the left twin would be able to be delivered vaginally	[22]
29	35	absent (removed in the past)	twin pregnancy	breech presentation	cesarean section	breech presentation	[23]
ND	30	present	twin pregnancy	ND	natural childbirth	N/A	[24]
36	36	ND	twin pregnancy	ND	cesarean section	chorio-amnionitis	[25]
26	34	ND	left	breech presentation	cesarean section	breech presentation	[26]
ND	26	present	twin pregnancy	ND	natural childbirth (delayed delivery between twins)	ND	[27]
ND	30	present	right	occiput posterior position	natural childbirth	N/A	[28]
18	23	absent (removed in the past)	left	anterior presentation	natural childbirth	N/A	[29]
ND	36	ND	right	cephalic presentation	cesarean section	status after two cesarean sections	[30]
25	25	ND	right	breech presentation	cesarean section	primigravida, breech presentation with suspected intra-hepatic cholestasis of pregnancy and uterine malformation	[31]
20	20	present	ND	ND	cesarean section	risk of rupture of a scar from a previous pregnancy	[32]
19	19	present	ND	N/A	case with 9 weeks of gestation underwent manual vacuum aspiration (MVA)	N/A	[32]
37	37	present	twin pregnancy	cephalic presentation	cesarean section	the fetus in the uterus with the rarely dilated vagina was expected to present a difficult delivery, an unpredictable labor course might occur arising from the competition of two simultaneously laboring uteruses	[33]
ND	34	absent	twin pregnancy	ND	natural childbirth	N/A	[34]
ND	28	absent (removed in the past)	ND	breech presentation	cesarean section	breech presentation	[35]
28	30	absent	ND	cephalic position	natural childbirth	N/A	[36]
22	22	present	left	ND	spontaneous abortion	ND	[37]

## Data Availability

No new data were created or analyzed in this study. Data sharing is not applicable to this article.
